# A 3D Bioprinted Pancreatic Cancer Model Using Collagen-Gelatin Methacrylamide-Alginate Bioinks to Mimic the Desmoplastic Microenvironment

**DOI:** 10.1021/acs.biomac.5c00450

**Published:** 2025-10-14

**Authors:** Uxia Gato-Diaz, Sandra Blanco-Garcia, Diana Peixoto, Angel Concheiro, Carmen Alvarez-Lorenzo, Barbara Blanco-Fernandez

**Affiliations:** 1 I+D Farma Group (GI-1645), Department of Pharmacology, Pharmacy and Pharmaceutical Technology, Facultad de Farmacia and Instituto de Materiales (iMATUS), Universidade de Santiago de Compostela, Santiago de Compostela 15782, Spain; 2 Health Research Institute of Santiago de Compostela (IDIS), Complexo Hospitalario Universitario de Santiago de Compostela, Travesa da Choupana s/n., Santiago de Compostela 15706, Spain; 3 Department of Pharmaceutical Technology, Faculty of Pharmacy of the University of Coimbra, University of Coimbra, 3000-548 Coimbra, Portugal; 4 REQUIMTE/LAQV, Group of Pharmaceutical Technology, Faculty of Pharmacy of the University of Coimbra, University of Coimbra, 3000-548 Coimbra, Portugal

## Abstract

Pancreatic ductal adenocarcinoma is one of the cancers with the least favorable survival prognosis worldwide. It is characterized by a high desmoplastic stroma rich in collagen I, which regulates pancreatic cancer cells’ behavior. There is a critical need to develop desmoplastic 3D models for preclinical testing. In this study, bioinks that imitate the biochemical characteristics of pancreatic ductal adenocarcinoma were developed to observe the influence that the desmoplastic extracellular matrix has on cancer cells. The bioinks were made of gelatin methacrylamide, alginate, and different concentrations of collagen I. Cancer cells were able to proliferate in all bioinks, presenting high paclitaxel resistance and a high expression of desmoplasia and extracellular matrix remodeling markers. The designed bioinks can play a crucial role in developing more clinically relevant cancer models for chemotherapeutic drug screening. Furthermore, they have significant potential for studying the influence of desmoplasia and for improving advanced treatment approaches for pancreatic cancer.

## Graphical abstract

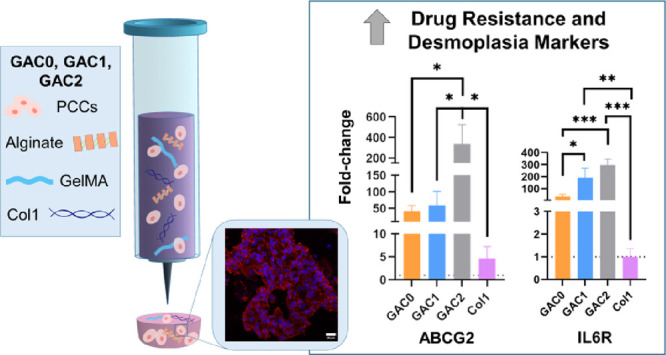

## Introduction

Pancreatic cancer is among the most aggressive cancers, with a five-year survival rate of less than 10%.^1–3^ Most pancreatic tumors originate from the exocrine tissue, with pancreatic ductal adenocarcinoma (PDAC) being the most prevalent and aggressive subtype.^4,5^ The asymptomatic nature of this disease in its early stages often delays diagnosis and treatment. Furthermore, conventional therapies such as chemotherapy and radiation therapy show limited efficacy in advanced stages due to the tumor’s characteristics. These factors position PDAC as one of the leading causes of cancer-related mortality worldwide.^4–6^ The high resistance of PDAC is due to the unique composition and behavior of its tumor microenvironment (TME), which serves as both a physical and immunosuppressive barrier.^6,7^

The PDAC TME consists predominantly of cancer cells, cancer-associated fibroblasts (CAFs), immune cells, and the extracellular matrix (ECM). During tumor development, the TME suffers significant alterations to facilitate tumor growth and drug resistance.^6,8,9^ A defining characteristic of this type of tumor is desmoplasia, marked by a dense and fibrous stroma. Desmoplasia is driven by the transition of fibroblastic-type cells into a myofibroblastic phenotype and an abnormal ECM deposition by CAFs.^9,10^ The high buildup of ECM proteins, including collagen I (Col1), fibronectin, and hyaluronic acid, within the tumor serves as a direct physical barrier, limiting the penetration of therapeutics.^11^ Furthermore, the high density of the stroma increases intratumoral pressure and compresses blood vessels, impairing drug delivery and disrupting the enhanced permeability and retention effect.^7^ All these changes that occur in the PDAC ECM during tumor development also have an influence on pancreatic cancer cell (PCC) behavior. Through different receptors, such as integrins, PCCs interact with different ECM elements, such as collagens or fibronectin.^12^ These molecules are able to induce changes in PCC migration, invasion, and adhesion, thereby modulating PDAC behavior and outcome.^13^

The desmoplastic TME in PDAC contributes to the reduced efficacy of cancer treatments and hinders the development of more effective drugs in preclinical studies.^13^ The most used preclinical models remain 2D in vitro and animal models. However, these models fail to accurately replicate the physiological characteristics of in vivo PDAC, limiting their translational relevance to clinical trials.^14^ Replicating TME characteristics and cell-ECM interactions is essential for developing relevant preclinical cancer models. Three-dimensional (3D) in vitro cancer models offer a more predictive alternative, as they can mimic the tumor architecture and ECM, and the cell interactions and behavior.^15,16^ This is supported by previous studies demonstrating that hydrogels that mimic the tumor ECM with decellularized ECM and that replicate certain PDAC architectural features can replicate many metabolomic pathways occurring in vivo.^17^ Therefore, scaffold-based 3D models, consisting of a matrix composed of ECM elements, can provide the biochemical and mechanical signals essential to support tumor progression, invasion, and metastasis.^15,18–20^

The need for more relevant and translational PDAC models for drug screening has driven the development of 3D models that more accurately replicate human TME. Among these, 3D bioprinting is an advanced biofabrication technique that enables the creation of highly precise and complex 3D models.^21,22^ For instance, bioprinted models incorporating various cell types from the PDAC and reproducing the TME architecture have been shown to effectively mimic in vivo cellular responses and behavior.^23,24^ Despite the advances suggested by these studies, the development of 3D printed PDAC ECM-based bioinks that effectively replicate the desmoplasia observed in these tumors remains underexplored.^25–27^ Further research is needed to investigate the influence of desmoplasia and Col1 on cellular behavior.

The primary aim of this study is to develop a 3D bioprinted PDAC model by microextrusion capable of mimicking tumor desmoplasia for screening of chemotherapy agents. The strategy employed to replicate the desmoplastic microenvironment involves the use of bioinks based on Col1, highly secreted in desmoplastic PDAC tumors.^28^ Indeed, Col1 also influences PCCs, enhancing their malignant behavior and, consequently, increasing the physiological relevance of the models for in vitro studies.^29^ Two rheological modifiers were also added to the bioinks to ensure their printability and the replication of the tumor stiffness: gelatin methacrylamide (GelMA) and alginate. GelMA addition enhances the rheological properties of the bioinks, enabling bioink printability while mimicking some of the 3D characteristics of the native ECM. Meanwhile, alginate increases the hydrogel rigidity, making the model more comparable to tumor tissues. In this work, bioink characterization was conducted to assess the mechanical properties, printability, and porosity of the bioinks and hydrogels. Moreover, the designed hydrogel models were evaluated to assess the similarities between the PCCs embedded within them and those embedded in in vivo tumors. This analysis was done by evaluating cell viability, proliferation, morphology, and PCC's expression of malignancy and drug resistance markers. Overall, these 3D bioprinted PDAC models offer a powerful new approach for studying tumor-ECM interactions and testing potential new treatments. By closely mimicking the tumor ECM, they provide a valuable tool for advancing drug screening and improving our understanding of pancreatic cancer.

## Experimental Section

### Materials

Alginate (100,000–200,000 g/mol, ref. 71238), bovine serum albumin (BSA), calcium chloride (CaCl_2_), gelatin (type A, ∼300 g of Bloom, ref. G1890), lithium phenyl-2,4,6-trimethylbenzoylphosphinate (LAP), and phalloidin–tetramethylrhodamine B isothiocyanate (ref. P1951), RT-qPCR primers (sequences in Table S1), sodium hydroxide (NaOH), Triton X-100, and 4′,6-diamidino-2-phenylindole (DAPI) were acquired from Sigma-Aldrich (St. Louis, MO, USA). Antibiotic-antimycotic solution (ref. 15240062), Dulbecco’s phosphate-buffered saline (DPBS) 10x, fetal bovine serum (FBS, ref. 10270106), *N*-(2-hydroxyethyl)­piperazine-*N*′-(2-ethanesulfonic acid) (HEPES), paraformaldehyde (PFA), phosphate-buffered saline tablets (ref. 18912014), and RPMI 1640 supplemented with Glutamax (ref. 61870036) were purchased from Thermo Fisher Scientific (Waltham, MA, USA). Paclitaxel was purchased from Thermo Scientific Alfa Aesar (Karlsruhe, Germany). Col1 was prepared as previously reported.^30^

### Cell Culture

Pancreatic cancer cells (BxPC-3, CRL-1687, ATCC) were cultured in RPMI 1640 supplemented with Glutamax, 10% fetal bovine serum, and 1% antibiotic–antimycotic. The cell medium was changed every other day.

### Bioinks Preparation

Three bioinks were prepared with different concentrations of Col1 (Table 1): 0 (GAC0), 1.5 (GAC1), and 3 mg/mL (GAC2). GelMA was synthesized as previously reported by our group,^31^ and the degree of functionalization was evaluated by ^1^H NMR (Figure S1). Then, GelMA was dispersed in complete cell medium at 37 °C, while the alginate dispersion and LAP solution were prepared in PBS. The required volumes of alginate and cell medium preheated at 37 °C were added to the GelMA dispersion, followed by further stirring and cooling to room temperature. Next, the Col1 dispersion was neutralized with 1 M NaOH, its osmolarity was adjusted with 10x DPBS to a final concentration of 1x, and it was added to the biopolymer dispersions with LAP. Finally, BXPC-3 cells dispersed in the cell medium were incorporated into the bioink to achieve a final cell density of 3·10^6^ cells/mL, and cell media were added to achieve the final polymer concentrations specified in Table 1.

**Table 1:** Composition of Each Bioink and Conditions Used during Bioprinting

component	GAC0	GAC1	GAC2
GelMA (%)	5	5	5
alginate (%)	0.5	0.5	0.5
LAP (%)	0.2	0.2	0.2
collagen I (%)^a^	0	0.15	0.3
cell media (%)	q.s.	q.s.	q.s.
cell density (cell/mL)	3·10^6^	3·10^6^	3·10^6^
bioprinting temperature (°C)	24	24	24
pressure (Bar)	0.3	0.3	0.5
speed (mm/s)	15	22	22
platform temperature (°C)	15	15	15

a NaOH 1 M and DPBS10X were added to the collagen I dispersion to have neutral pH and to achieve an osmolarity compatible with cell viability.

### Bioink Characterization

The mechanical properties, printability, and porosity of the bioinks (without cells) were assessed. The Young's modulus was assessed by compression studies using a texture analyzer (TX plus, Texture Technologies, Hamilton, MA, USA) equipped with a 5 N load cell and a 20 mm cylindrical aluminum probe. To prepare the specimens, bioinks (500 μL) were cross-linked in 48-well plates with blue light (405 nm, 1 min) and 50 mM CaCl_2_ in 10 mM HEPES (200 μL, 10 min), followed by washing with PBS. Then, an additional 100 μL of PBS was added to each gel, and the cells were incubated at 37 °C overnight. Young’s modulus was calculated by determining the slope from a graph of strain versus applied force. Four specimens were prepared for each condition.

Bioinks’ rheological characteristics were recorded, in triplicate, in a MCR302 rheometer (Anton Paar, Graz, Austria) fitted with a 15 mm diameter plane geometry and a gap of 1 mm at 24 °C. The storage (*G*′) and loss (*G*″) moduli were measured during five cycles of shear strain recreating the resting conditions in the printer cartridge (0.5% shear stress at 1 Hz, 120 s), the extrusion conditions (100% shear strain at 1 Hz, 300 s), resting (0.5% shear stress at 1 Hz, 120 s), extrusion (100% shear strain at 1 Hz, 300 s), and resting (0.5% shear stress at 1 Hz, 120 s).

Hydrogel porosity was assessed by scanning electron microscopy (SEM, ZEISS FESEM Ultra Plus, Zeiss, Jena, Germany) after being freeze-dried and sputter-coated with iridium (10 nm, Quorum Q150T-S-Plus, Quorum Technologies).

The printabilities and shape fidelities of GAC0, GAC1, and GAC2 bioinks were evaluated with a Bioplotter Manufacturer (EnvisionTEC, Gladbeck, Germany). Initially, the formation of filaments during extrusion under the defined printing conditions for each bioink was visually verified. To evaluate the printing fidelity, five filaments of 16 mm in length were printed, and the diameter (at 30 different positions) and length were measured using FIJI (Figure S2a).^32^ The spreading ratio was calculated by dividing the diameter of the printed filament by the inner diameter of the needle. Additionally, scaffolds with varying pore sizes (ranging from 2 × 2 mm to 5 × 5 mm) were fabricated to evaluate the printability of the bioinks. The area and perimeter of the pores were measured, and the printability and diffusion rate were determined using eqs 1 and 2.

$$\mathrm{printability}=\frac{L^{2}}{16xA_{\exp}}$$ 1

$$\mathrm{diffusion}\;\mathrm{rate}=\frac{A_{t}-A_{\exp}}{A_{t}}x100$$ 2

where *L* is the pore’s perimeter, *A*_exp_ is the pore’s area, and *A*_t_ is the theoretical pore’s area.

### Tumoroids Bioprinting

Tumoroids were designed with Sketchup (Figure S2b), and hydrogels were printed using a Bioplotter Manufacturer (EnvisionTEC, Gladbeck, Germany) in 24-well plates. BxPC-3 cells were resuspended in the bioinks at a cell density of 3·10^6^ cells/mL, and bioinks were loaded into print cartridges and extruded through a 21 G (0.51 mm diameter) needle. The extrusion of the bioinks was carried out at 24 °C with pressures between 0.3 and 0.5 bar and extrusion speeds of 15–22 mm/s (Table 1). Then, hydrogels were cross-linked with visible light (405 nm, 60 s), CaCl_2_ (50 mM in HEPES 10 mM, 10 min), and by incubating the cell-laden hydrogels at 37 °C for 30 min. Finally, 0.5 mL of complete cell media was added to each well, and cell-laden hydrogels were kept for up to 9 days in culture. Cell media was replaced every other day. 2D controls (75,000 cells) and 3D controls using Col1 at 4 mg/mL (25 μL) were also prepared. Col1 dispersion was neutralized with NaOH 1M, and its osmolarity was corrected with DPBS 10x. Then, it was diluted with the complete cell medium to have a final concentration of 4 mg/mL. Finally, Col1 at 4 mg/mL was added to a cell pellet to have a final cell density of 3·10^6^ cells/mL, and 25 μL gels were prepared in a 24-well plate. The gels were cross-linked for 20 min in an incubator at 37 °C, and 0.5 mL of complete cell media was added.

### Cell Viability

Cell viability was assessed through a LIVE/DEAD assay (ref. L3224, ThermoFisher Scientific, Waltham, MA, USA) following the manufacturer’s instructions. On days 1 and 7, cell-laden hydrogels were washed with DPBS 1X once, incubated with calcein AM (2 μM) and ethidium homodimer-1 (4 μM) for 20 min (*n* = 3), and washed again with DPBS 1X. Live and dead cells were imaged with a confocal microscope (Stellaris, Leica Microsystems). The cellular viability was quantified on day 1 with a 3D object counter of FIJI.^32^

### Cell Proliferation

Cell proliferation in the bioprinted hydrogels was assessed by measuring both the metabolic activity and DNA content. The cellular metabolic activity in the bioprinted hydrogels was assessed using AlamarBlue (ref. DAL1025, ThermoFisher Scientific, Waltham, MA, USA). On days 1, 2, 3, 5, 7, and 9, the cell culture medium was replaced with AlamarBlue (10% in complete cell medium). After 1 h of incubation, the fluorescence was measured at 540/580 nm using a plate reader (FLUOstar Optima, BMG Labtech Microplate Readers, Ortenberg, Germany). Five replicates per condition were analyzed. 2D and Col1 hydrogels were used as controls.

The double-strand DNA (dsDNA) was quantified with a Quant-iT PicoGreen kit (ref. P7581, ThermoFisher Scientific, Waltham, MA, USA). On days 0, 3, 7, and 9, cell-laden hydrogels were washed with PBS and frozen with 500 μL of TE buffer (*n* = 4). Then, samples were subjected to three freeze–thaw cycles, and hydrogels were mechanically disrupted with the help of a needle (21G) and a syringe. Finally, the samples were spun at 10,000*g* for 5 min, and the supernatant was used for the dsDNA quantification according to the manufacturer’s instructions at 485/520 nm. Four replicates per condition were analyzed.

### Cell Staining

For visualizing cell distribution and morphology, cell-laden hydrogels were stained with phalloidin/DAPI (*n* = 3). Cell-laden bioprinted hydrogels were first washed once with PBS, then fixed with paraformaldehyde (4%, 20 min, RT), and permeabilized with 0.1% Triton X-100 (5 min, RT). Afterward, the samples were incubated with phalloidin–tetramethylrhodamine B isothiocyanate (50 μg/mL, 45 min, RT) and DAPI (1 μg/mL, 10 min, RT). Between each step, the hydrogels were washed three times with PBS. Cells were visualized using a confocal microscope (Stellaris, Leica Microsystems). Nonbioprinted hydrogels made of Col1 at 4 mg/mL were used as controls.

### Drug Response

To assess the efficacy of chemotherapeutic drugs, paclitaxel was evaluated against cell-laden bioprinted hydrogels. Bioprinted hydrogels (*n* = 4) and Col1 controls (*n* = 5) were cultured for 7 days to ensure the growth of BxPC-3 cells in the hydrogels, whereas in 2D controls (*n* = 6), BxPC-3 cells were seeded in 24-well plates and incubated overnight. The hydrogels and 2D controls were then exposed to varying concentrations of paclitaxel for 48 h. Paclitaxel solutions (0.01, 0.1, 1, 10, and 100 μM) were prepared in the complete cell medium using a stock solution of 10 mM paclitaxel in DMSO. Cell viability was assessed using AlamarBlue as described in Cell Proliferation. Negative controls were prepared in the cell medium containing the same concentration of DMSO at each paclitaxel concentration. IC50 values were determined by using Prism 8.0 (GraphPad Software).

### Real-Time Quantitative Polymerase Chain Reaction (RT-qPCR)

The differences in the gene expression were evaluated by RT-qPCR (*n* = 3). Bioprinted cell-laden hydrogels and 2D and Col1 controls were cultured for 7 days, washed with PBS once, and collected into RLT lysis buffer with 1% β-mercaptoethanol (350 μL). Samples were stored at −80 °C until the RNA isolation. To isolate the RNA, samples were homogeneously disrupted by three cycles of freeze–thaw and mechanical disruption with the help of a needle (21G) and syringe. RNA was isolated with the RNeasy Plus Mini Kit (ref 74134, Qiagen, Hilden, Germany), and its concentration was quantified with a UV/vis nano spectrophotometer (Nabi, Microdigital, Granada, Spain). Then, cDNA was synthesized by using the iScript cDNA Synthesis Kit (BIO-RAD, Hercules, CA, USA). RT-qPCRs were performed by mixing the synthesized cDNA (1.5 ng), iTaq Universal SYBR Green Supermix (BIO-RAD, Hercules, CA, US), and the corresponding primer (500 nM, Table S1) and run in a QuantStudio 3 system (Applied Biosystems, Waltham, MA, USA). The RT-qPCR conditions were 1 cycle of 10 min at 95 °C and 40 cycles of 15 s at 95 °C and 1 min at 60 °C, followed by the melting curves. The calculations of the gene expression fold change were carried out with the 2^–ΔΔCt^ method, with β-actin serving as the housekeeping gene.

### Statistical Analysis

Statistical analyses were performed using GraphPad Prism 8.0 (GraphPad Software). Data were analyzed using *t* tests or one-way or two-way ANOVA, as appropriate, to assess differences between conditions. A *p*-value of less than 0.05 was considered statistically significant. The data points presented in the charts represent the mean ± the standard deviation (SD) of the measurements.

## Results and Discussion

### Bioinks and Hydrogel Characterization

Fibrillar collagens, primarily Col1 and collagen III, are present in most tumor types and are synthesized by CAFs and tumor cells.^33–35^ In the case of PDAC, these collagens are the most abundant proteins, contributing to the tumor fibrosis characteristic of desmoplasia.^36,37^ The role of Col1 in PDAC is not yet fully understood, but studies suggest that it significantly influences PCC behavior. Col1 has been implicated in promoting metastasis and increasing cell motility, enhancing the secretion of ECM remodeling enzymes such as matrix metalloproteinases (MMPs), contributing to drug resistance, and activating intracellular signaling pathways through mechanical stimuli.^38–40^ For this reason, a bioink based on Col1 was developed to replicate the malignant cell responses observed in tumors under in vivo conditions.

Col1, a naturally derived hydrogel, is widely used in 3D printing applications.^41^ However, it presents certain challenges for extrusion-based bioprinting. Col1 remains in a liquid state at low temperature, and upon exposure to higher temperatures at neutral pH, it forms a fibrous structure. Its slow gelation process allows gravity to cause uneven cell distribution, resulting in nonuniformity within the construct. Furthermore, its low mechanical strength and instability underscore the necessity for rheological modifiers to improve the properties of bioinks.^41,42^ The rheological modifiers utilized in the development of our bioinks were GelMA and alginate. GelMA exhibits suitable rheological properties for extrusion, can be easily combined with other materials, cross-links rapidly under UV/blue light to minimize cell sedimentation, and supports cell proliferation and migration.^42,43^ In this study, GelMA was produced following previous reports,^31^ obtaining a functionalization level of 62.9% (Figure S1). Alginate is widely used in bioinks due to its ability to enhance stiffness and regulate the viscosity and porosity of the bioinks. The combination of both GelMA and alginate enables us to overcome many of the limitations they exhibit when used independently.^42,43^

The bioinks’ rheological properties were analyzed to elucidate their viscoelastic behavior under bioprinting conditions.^44^ During 3D printing, bioinks experience high shear stress as they are extruded through the nozzle and should rapidly recover their storage and loss moduli once the stress is released. To assess the bioinks’ response under extrusion, a cyclic sweep test was conducted using alternating low and high strains (Figure 1A).^44^ The *G*′ values of GAC0, GAC1, and GAC2 dropped sharply under high strain and instantaneously recovered in the low strain regime. The recoverability of each bioink showed minor differences between cycles, demonstrating their self-healing properties and suitability for 3D printing.^45^ GAC0 bioink transitioned from gel-like behavior at low strain (*G*′ > *G*″) to liquid-like behavior at high strain (*G*′ < *G*″) and recovered the gel-like behavior once the strain was reduced to its resting state (0.5%) in all cycles. Differently, bioinks containing Col1 showed similar values of *G*′ and *G*″ under high strain, indicating that Col1 increased the elastic response of the bioinks under high strain.

**Figure 1:**
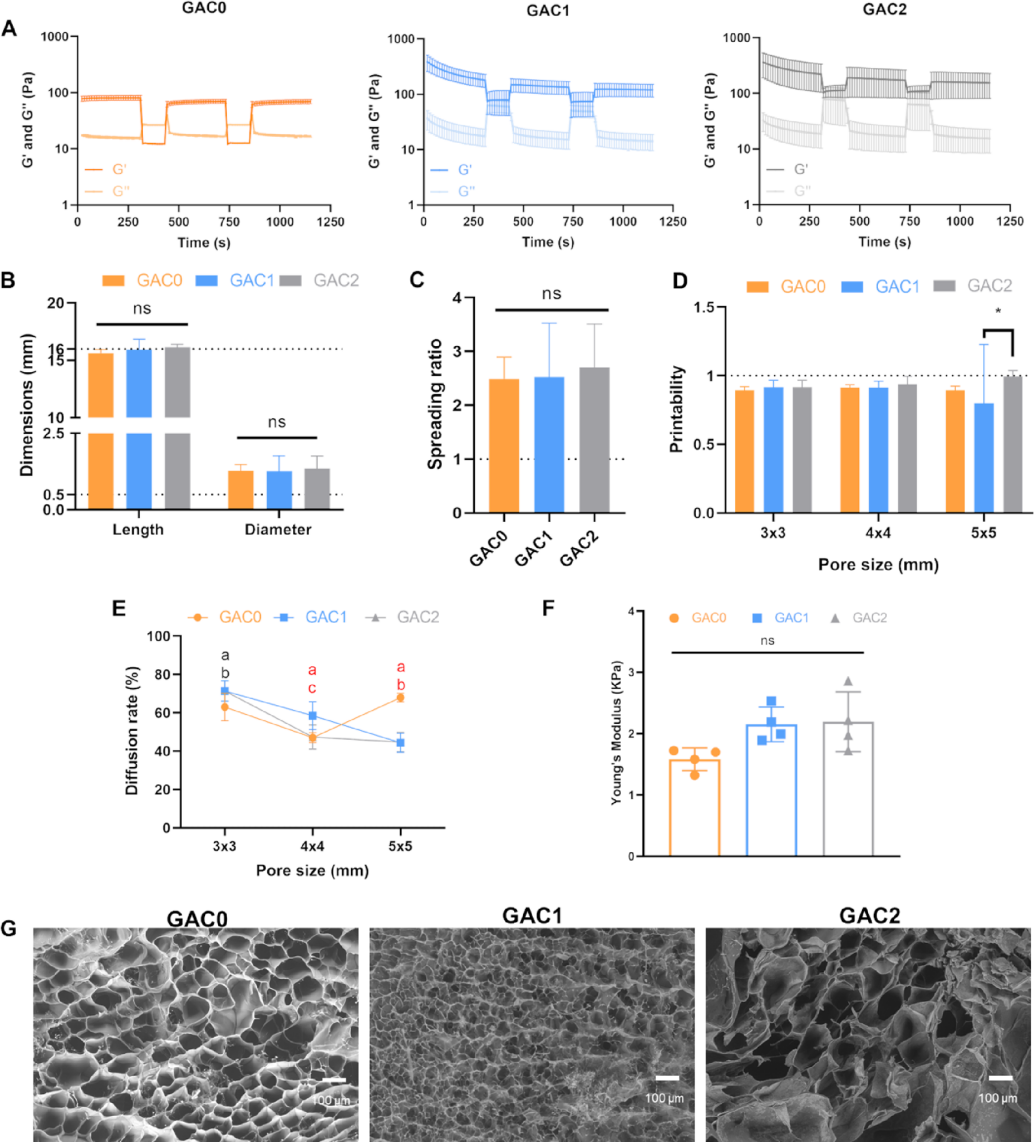
Printability, mechanical properties, and porosity of GAC0, GAC1, and GAC2 bioinks. (A) Rheological properties of the bioinks under cyclic changes of the shear strain to simulate the resting (0.5%) and bioprinting conditions (100%). (B–E) Printability and shape fidelity of the bioinks: bioprinted filaments length and diameter with a theoretical length of 16 mm and a diameter of 0.51 mm (B), spreading ratio (C), printability (D), and diffusion ratio (E). (F) Young’s modulus of the hydrogels. (G) SEM micrographs showing the porosity of the hydrogels (scale bar: 100 μm).

Bioinks’ printability was evaluated by measuring the diameter, the length, and the spreading ratio of the printed filaments. All bioink filaments exhibited diameters larger than the nozzle inner diameter (0.51 mm) and had a shorter length than the designed filament (16 mm, Figure 1B), with spreading ratios of approximately 2.5–2.7 (GAC0: 2.49 ± 0.40, GAC1: 2.52 ± 1.00, and GAC2: 2.70 ± 0.81; Figure 1C) with no differences among conditions. However, all values are acceptable to ensure a precise bioprinting process.^46^ The diffusion rate and printability of the bioinks were also determined by bioprinting of scaffolds with different pore sizes (Figure S3). At small pore sizes (≤2 mm × 2 mm), the filaments fused together, requiring theoretical pores ≥3 × 3 mm to form distinct pores. All bioinks showed printability values above 0.9, and only GAC2 at larger pore sizes achieved values close to 1 (Figure 1D). The presence of Col1 in the bioinks allowed a reduction of the diffusion rate at larger pores, indicating that its presence improved the shape fidelity (Figure 1E).

The Young’s modulus of the hydrogels was also determined to ensure that the stiffness of the bioprinted hydrogels replicated the stiffness of PDAC. The hydrogels exhibited a modulus ranging from 1.6 to 2.2 kPa, indicating their ability to mimic tumor stiffness (Figure 1F).^47^ Additionally, the presence of Col1 tended to increase the hydrogel stiffness. All hydrogels were highly porous, with no differences among conditions and indicating that the porosity could support the diffusion of nutrients, oxygen, and cell migration.

### Addition of Col1 into the Bioinks Supports Cell Proliferation

Previous studies have highlighted the role of Col1 in promoting PCC proliferation, migration, and antiapoptotic properties.^48,49^ Based on these findings, we investigated whether the bioinks based on Col1 would impact PCC growth. BxPC-3 cells were resuspended in the bioinks, and scaffolds with a cylinder shape (Figure S2B) were bioprinted. First, the PCC's viability in the bioprinted cell-laden hydrogels was evaluated by confocal microscopy and compared to a nonbioprinted 3D control based on Col1. On day 1, high cell viability could be detected under all conditions (≥70%, Figure 2A,B). Bioinks containing Col1 (GAC1 and GAC2) showed no differences in cell viability with the control, evidencing that the bioprinting conditions and bioink composition do not compromise cell viability. In the case of GAC0, the cell mortality rate was slightly higher than that in the control (Figure 2B), which might be due to the stress experienced by the cells during extrusion, as this bioink was less viscous. Nevertheless, on day 7, PCCs exhibited high viability with no differences among the conditions (Figure 2C). Therefore, all bioinks support the cell viability of PCCs over 1 week.

**Figure 2:**
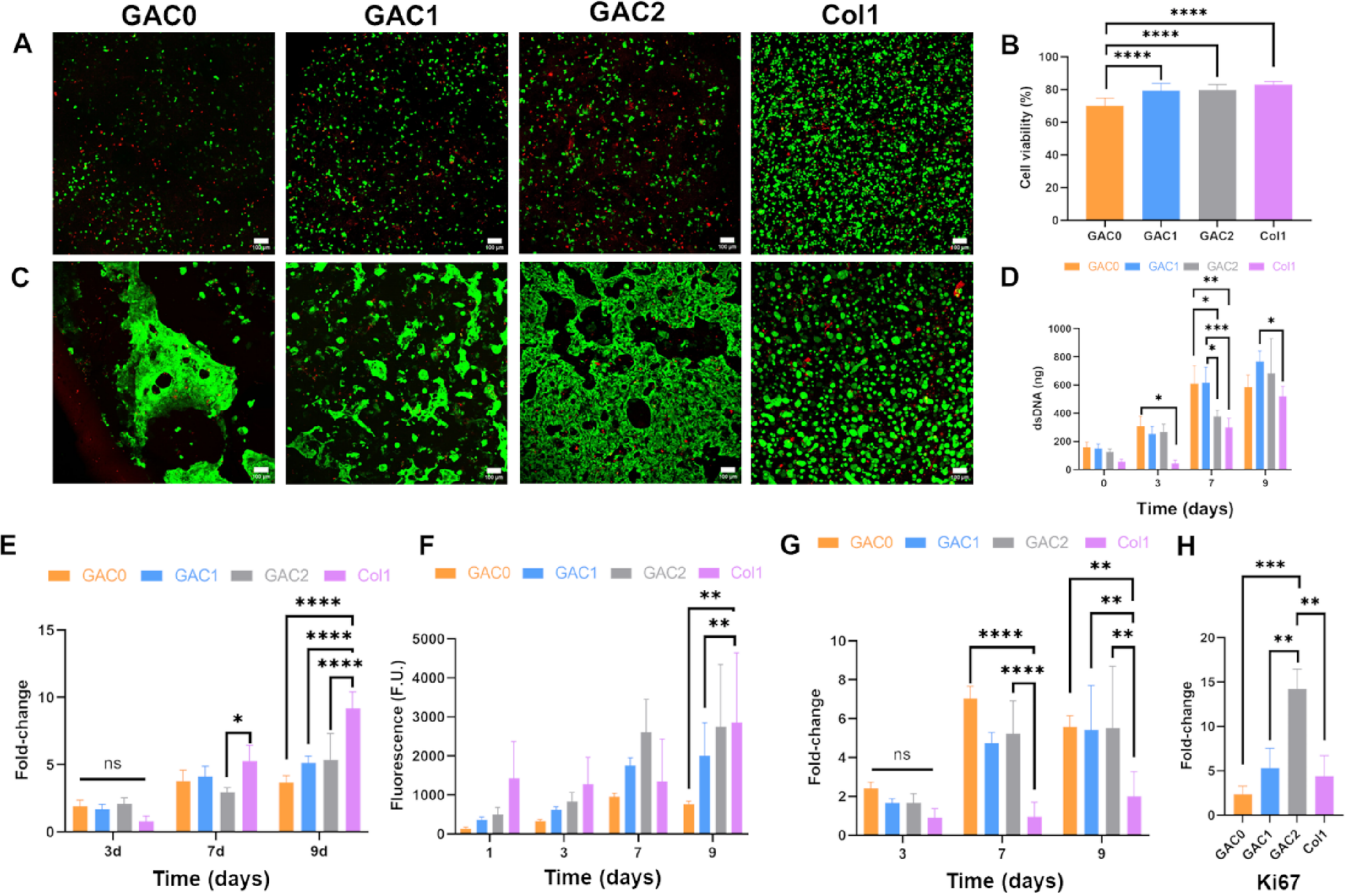
BxPC-3 viability and proliferation in cell-laden bioprinted hydrogel. (A, C) Cell viability after 1 day (A, B) and 7 days (C) in culture. (A, C) Alive cells were stained with calcein AM (green) and dead cells with ethidium homodimer-1 (red). Scale bar: 100 μm. (B) Quantification of alive cells on day 1. (D) dsDNA in the hydrogels after bioprinting and after 3, 7, and 9 days in culture (Two-way ANOVA, *n* = 5). (E) dsDNA values normalized by the amount of dsDNA after bioprinting (two-way ANOVA, *n* = 5). (F) Metabolic activity over time by AlamarBlue (two-way ANOVA, *n* = 4). (G) Metabolic activity normalized by day 1 (two-way ANOVA, *n* = 4). (H) Expression of the Ki67 gene by RT-qPCR (one-way ANOVA, *n* = 3). ns stands for no statistical difference, * *p* < 0.05, ** *p* < 0.01, *** *p* < 0.001, and **** *p* < 0.0001.

Subsequently, the effect of Col1 in the bioinks on PCC proliferation was evaluated by determining the dsDNA levels over time (Figure 2D). All bioinks showed a higher amount of dsDNA than the Col1 control, but only GAC0 and GAC1 showed statistically significant higher values after 1 week in culture. Additionally, both GAC0 and GAC1 showed higher quantities of dsDNA than GAC2. Nevertheless, to ensure comparability between conditions, the data were normalized to day 0, eliminating any variations in the initial PCC density (Figure 2E). No significant differences were observed between the bioprinted models and Col1 controls on day 3, but PCCs exhibited greater proliferation in the controls than the models, with no variation among conditions on days 7 and 9. These results may be a consequence of the stiffness differences observed between the models. The GAC0, GAC1, and GAC2 models are stiffer than the Col1 control, resulting in a proliferation inhibition similar to that produced by the desmoplastic stroma of PDAC.^50^ Consequently, the incorporation of Col1 into the bioinks imitates the desmoplastic reaction, inhibiting cellular proliferation.

The metabolic activity of PCCs was also monitored over time with AlamarBlue (Figure 2F). No statistically significant differences were observed between the bioprinted models and the Col1 control at days 1–7. However, Col1 control exhibited higher metabolic activity compared to GAC0 and GAC1 by day 9. To elucidate the small differences in viable cells under each condition, the results were normalized to day 1 (Figure 2G). All bioprinted models demonstrated higher metabolic activity rates than Col1 after 1 week in culture. However, no differences were observed between bioinks. Therefore, these results indicate that the incorporation of Col1 into the models does not enhance the metabolic activity of PCCs.

Finally, to gain a deeper understanding of proliferation, the expression of Ki67, a proliferation marker frequently linked to the clinical progression of PDAC, was analyzed. Elevated Ki67 expression is often indicative of poor prognosis.^51,52^ We observed a significantly higher expression of Ki67 in the GAC2 model compared to those of GAC0, GAC1, and Col1. This finding indicates that the incorporation of high Col1 proportions in the bioinks enhances the proliferation of PCCs. The effect of Col1 on enhancing PCC proliferation has been previously observed in other studies, further supporting our findings.^53,54^ This aligns with the typical behavior of solid tumors, where Col1 promotes proliferation via β-catenin activation and stimulates metabolic activity through pathways such as the phosphoinositide 3-kinase (PI3K)/protein kinase B (Akt) signaling pathway.^55,56^

### Col1-Based Bioinks Induced the Formation of Cell Clusters and Matrix Remodeling

Cell morphology and distribution were evaluated through phalloidin/DAPI staining by confocal microscopy. On day 1, cells were individually and homogeneously distributed in all conditions, revealing that the bioinks ensure an even cell homogenization without cell sedimentation during the bioprinting and cross-linking processes (Figure 3A). On day 7, cells proliferated in the hydrogels, forming irregular spheroids and cell clusters. In all bioinks, PCCs grew in clusters, following an island growth pattern, whereas in Col1 control, they formed a flatter and more extensive cellular network colonizing the full hydrogel, which could be due to the lower stiffness of the Col1 control (<1 KPa).^57^ The island pattern growth of BXPC-3 cells has already been observed in other studies^58–60^ (Figure 3B).

**Figure 3:**
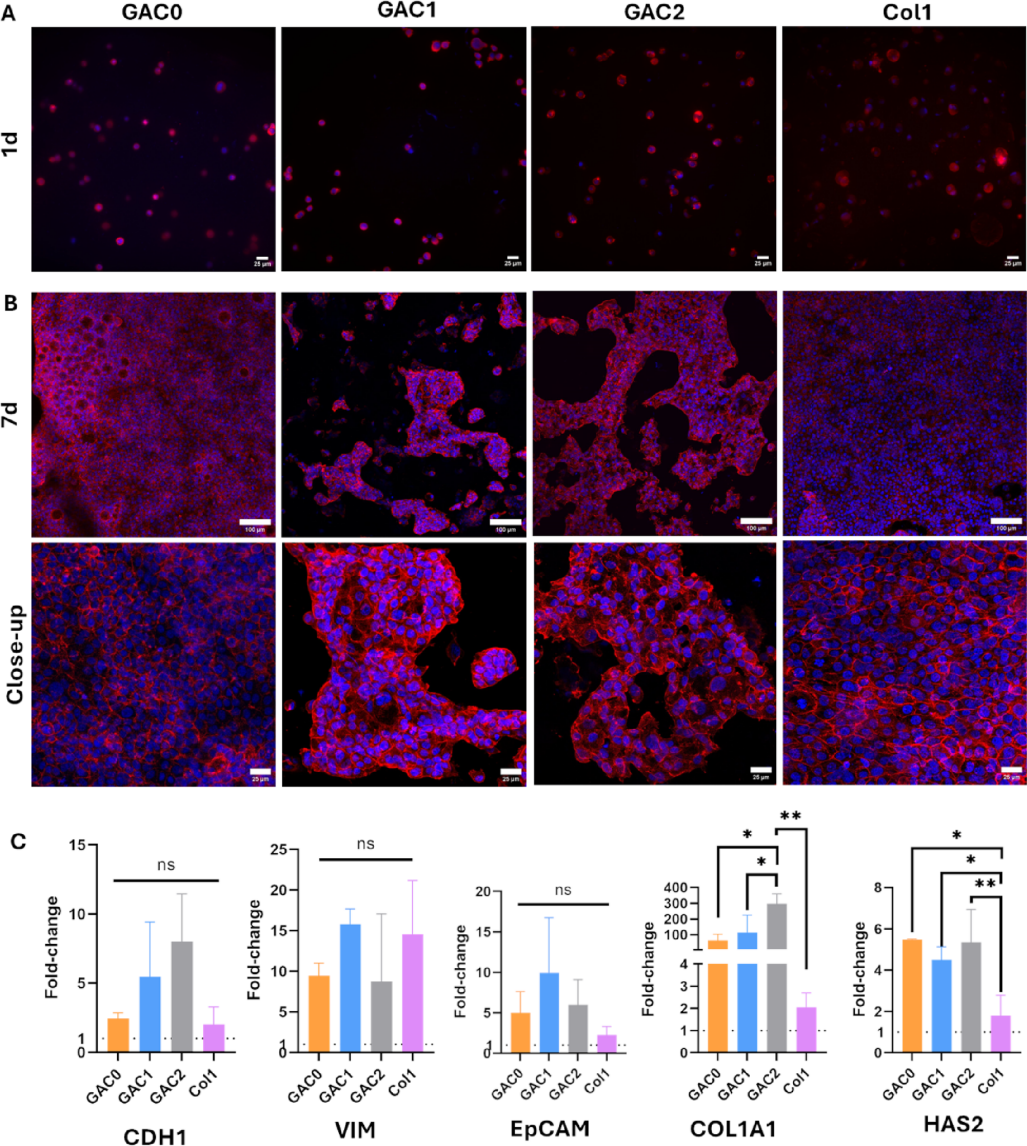
BxPC-3 morphology and expression of EMT and ECM remodeling markers in the bioprinted cell-laden hydrogels. (A, B) Cell morphology and distribution in the hydrogels after 1 day (A) and 7 days (B) in culture. The cytoskeleton was stained with phalloidin (red) and nuclei with DAPI (blue). Scale bars: 25 μm (A, B—close-up) and 100 μm (B—7d). (C) Expression of CDH1, VIM, EpCAM, COL1A1, and HAS2 in cells cultured for 7 days (one-way ANOVA, *n* = 3, ns: no significant, * *p* < 0.05, and ** *p* < 0.01). 2D cultures were used as controls to calculate the 2^–ΔΔCt^ values.

We then evaluated whether there were any differences in the expression of epithelial-mesenchymal transition (EMT) markers that could explain the cell distribution differences observed between the Col1 control and the bioprinted models (Figure 3C). We studied several markers related to the EMT process, including e-cadherin (CDH1), vimentin (VIM), and epithelial cell adhesion molecule (EpCAM). CDH1 is typically downregulated during EMT, which facilitates the increased invasiveness of PCCs.^50^ However, several studies have also indicated that the presence of Col1 can upregulate the expression of CDH1 in PDAC.^61^ In this study, a trend toward higher CDH1 expression was observed in GAC1 and GAC2, although no statistically significant differences were found between the models. VIM is another specific marker of EMT, expressed in the cytoplasm of mesenchymal cells.^62^ In this study, all models (GAC0, GAC1, and GAC2) and Col1 exhibited a trend toward high vimentin expression, but no statistically significant differences were found between them. EpCAM is a characteristic marker of epithelial cells that is downregulated during EMT.^50^ The designed models showed higher EpCAM expression, particularly in GAC1 and GAC2, but the differences were not statistically significant.

Then, we analyzed whether the bioinks were supporting hydrogel matrix remodeling by quantifying the expression of ECM markers, including collagen I (COL1A1) and hyaluronic acid synthase 2 (HAS2). PDAC desmoplasia is characterized by an abundant ECM, with Col1 and hyaluronic acid playing a crucial role in tumor progression and microenvironment remodeling.^63^ Previous studies have demonstrated that these two ECM components are overexpressed in PDAC tumors and are associated with a poor prognosis, contributing to disease progression and therapeutic resistance.^64^ Moreover, another study has demonstrated that Col1 coating of 3D scaffolds leads to dense desmoplasia and increases Col1 cell deposition.^19^ COL1A1 expression in our designed models tends to be higher than in the Col1 control, observing an overall increase in expression across all models. Notably, GAC2 exhibited a statistically significantly higher expression of this marker compared to the other models. In the case of HAS2, all bioprinted models presented a statistically higher expression compared to that of the Col1 control. Additionally, we analyzed the expression of fibronectin (FN1) and metallaproteinase IX (MMP9). However, no detectable expression was found in the GAC0, GAC1, and GAC2 models or in the Col1 control, with Ct values exceeding 35 in all cases.

The presence of Col1 in our models better replicates the ECM remodeling observed in PDAC tumors in vivo. Moreover, the small variations in ECM deposition between models could promote an increase in the stiffness of the tumoroid, which could explain the cell clustering observed in GAC0, GAC1, and GAC2 (Figure 3B).

### Presence of Col1 in the Bioinks Supports the Expression of Desmoplasia Markers

The next step was to investigate whether the inclusion of different concentrations of Col1 within the bioinks could influence the behavior of PCCs by enhancing the expression of key malignancy markers in PDAC and desmoplasia (Figure 4). The desmoplasia characteristic of PDAC is defined not only by a dense stroma but also by an inflammatory profile that influences PCC's invasiveness and malignancy. This prolonged inflammatory state fosters a microenvironment rich in cytokines and growth factors, which are released by various cellular components of the TME.^65^ Moreover, the PDAC desmoplasia interacts with stromal cells, promoting their malignant transformation, and with PCCs, inducing malignancy signals that regulate their behavior and gene expression.^28^ In particular, Col1, overexpressed in desmoplasia, is known to interact with cancer cells, activating various signaling pathways that regulate and enhance their malignant behavior.^66^ Additionally, the characteristics conferred by Col1 to the tumor, such as the mechanical stress, play a role in modulating the expression of malignancy markers in cancer cells.^66,67^ Col1 also activates integrin signaling, which leads to cytokine release, helping to maintain the inflammatory state of PDAC.^68^ Even though CAFs are the primary producers of cytokines, PDAC cells can also release interleukins and growth factors. These molecules contribute to their survival and promote malignant transformation.^69,70^

**Figure 4:**
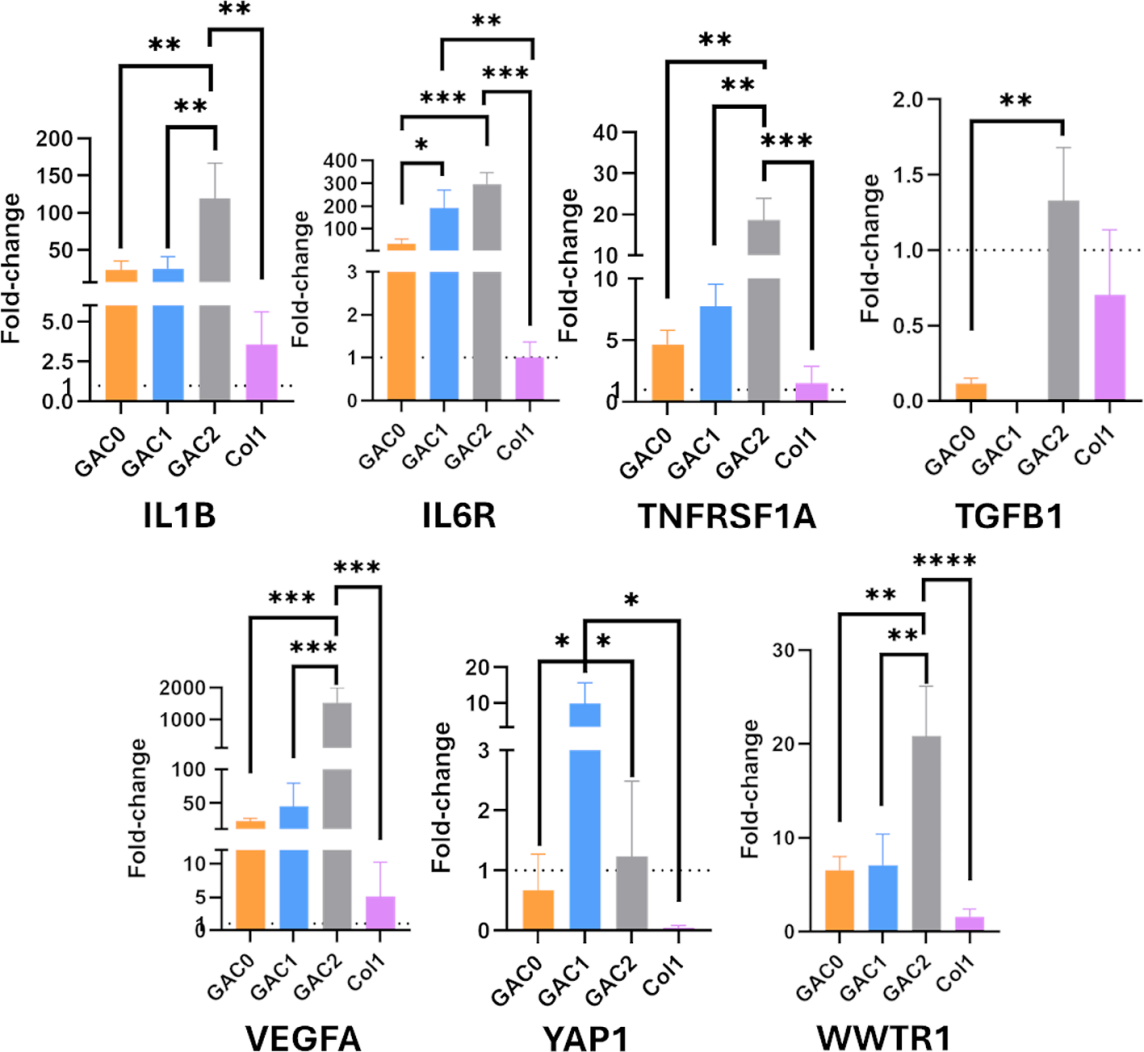
Expression of malignancy markers by BxPC-3 cultured in the bioprinted hydrogels and in Col1 controls for 7 days. IL1β, IL6R, TGFB1, TNFRSF1A, VEGFA, YAP1, and WWTR1 gene expression is expressed as 2^–ΔΔCt^ using 2D cultures as controls. (One-way ANOVA, *n* = 3, * *p* < 0.05, ** *p* < 0.01, and *** *p* < 0.001).

Macrophages, CAFs, and PCCs are the main producers of inflammatory cytokines within the TME. Some inflammatory signaling molecules, such as interleukin-1β (IL1β), interleukin-6 (IL-6), and tumor necrosis factor (TNF), play a crucial role in modulating the TME and influencing cancer progression.^71^ IL1β is involved in the EMT and metastasis in PDAC, and the overexpression of IL1β activates signaling pathways such as NFkB, IRAK4, and MAPK, which are associated with a poor disease prognosis.^70,72,73^ All bioinks enhanced the IL1β gene expression compared to the control, particularly the bioink containing a higher Col1 concentration (GAC2). We also assessed the expression of the receptors that bind IL-6 and TNF. The IL-6 receptor (IL6R) responds to IL-6, activating immune-suppressive signals and modulating the response to chemotherapy. The chronic presence of IL-6 in tumors sustains inflammation and is linked to cachexia and impairments in immune responses.^69,74,75^ On the other hand, TNF binds to its receptor (TNFRSF1A), triggering multiple signaling pathways involved in cell survival, inflammation, and pro-tumorigenesis. TNFRSF1A represents a potential therapeutic target for PDAC, as it promotes tumor growth, modulates interactions with immune cells, and contributes to immunosuppression.^76,77^ The bioprinted tumoroids containing Col1 (GAC1, GAC2) promoted the overexpression of both genes to a higher extent than the Col1 control and the bioink without Col1 (GAC0). The results obtained indicate that the addition of Col1 to the models enhances the mimicry of PDAC-associated chronic inflammation, leading to PCC behavior that more closely resembles that observed in vivo. Specifically, GAC2, the model with the highest concentration of Col1, exhibits the greatest expression of these inflammatory markers, further highlighting its potential as a robust preclinical PDAC model.

The transforming growth factor-β (TGFβ) is also linked to the desmoplasia signature by stimulating the differentiation of fibroblasts into CAF and enhancing the deposition of Col1 to produce the fibrotic TME.^78,79^ Additionally, this factor is also an immunosuppressive factor when overexpressed in the late stages of PDAC. Due to mutations in p53 in PCCs, TGFβ acts as a tumor promoter, contributing to cancer progression and metastasis.^80,81^ Our results indicate that the GAC2 model exhibits a higher expression of TGFβ compared with the other models. However, this difference is not statistically significant compared to the Col1 control.

Angiogenesis is a hallmark of PDAC progression, with the vascular endothelial growth factor (VEGF) being the predominant angiogenic factor. Secreted by PCCs and other stromal cells, VEGF promotes tumor invasiveness, metastasis, and neovascularization. Due to its critical role, VEGF serves as a key therapeutic target, and antiangiogenic therapies have been shown to effectively reduce tumor growth.^82,83^ Analysis of VEGF expression in the designed models revealed that the GAC2 model exhibits a statistically significantly higher expression compared to the other models and the Col1 control. This suggests that the addition of Col1 enhances the simulation of angiogenesis observed in vivo, thereby increasing the physiological relevance of these models.

PDAC desmoplasia is also characterized by a dense stroma that generates mechanical signals to PCCs, triggering the activation of mechano-sensing pathways. YAP1 and WWTR1 encode the transcriptional coactivators YAP and TAZ, respectively, whose expression is directly influenced by the mechano-transduction signals generated within the TME. In PCCs, YAP/TAZ activation plays a crucial role in promoting cell proliferation, particularly in advanced and metastatic stages of PDAC, and contributes to the development of drug resistance.^84,85^ In this study, increased expression of YAP1 in GAC1 and WWTR1 in GAC2 was observed, suggesting that the presence of Col1 in the designed models enhances mechanical signaling in PCCs, thereby improving the mimicry of the in vivo behavior. We hypothesize that the differences observed between the bioinks could be related to the ECM remodeling occurring during the tumoroid maturation (Figure 3C), as no differences in stiffness were observed between the bioinks.

All of these results suggest that our Col1-enriched models (GAC1 and GAC2) have the potential to serve as 3D PDAC models, as they effectively replicate the expression of key markers associated with the inflammatory and mechanotransductive microenvironment characteristic of desmoplasia. These findings highlight the preclinical relevance of these models, demonstrating their potential as effective tools for PDAC research.

### Incorporation of Col1 Increases the Resistance to Chemotherapy Agents

The aggressive nature of PDAC presents a significant challenge for effective treatment. Desmoplasia and hypoxia work synergistically, promoting PCC chemoresistance and preventing drug penetration into the TME.^40,86,87^ The incorporation of Col1 into our 3D models could effectively mimic the impact of desmoplasia, modulating PCC behavior and influencing their response to chemotherapeutic agents.

To evaluate the drug resistance of PCCs embedded within our models, we assessed the cell viability at varying concentrations of paclitaxel. We selected paclitaxel for this study because its albumin-bound formulation, in combination with gemcitabine, is considered a first-line treatment for metastatic PDAC.^88^ We compared BXPC-3 cell viability in GAC0, GAC1, and GAC2 models along with Col1 and 2D controls. After 7 days of incubation, paclitaxel was added to the models and controls for 48 h, followed by the assessment of cell viability. When analyzing the results (Figure 5A), we observed that the 2D control exhibited a lower IC50 (2D: 0.0098 μM) compared to the 3D models (GAC0: >100 μM, GAC1: 85.99 μM, GAC2: >100 μM, and Col1: 0.33 μM). Furthermore, we observed that all bioprinted models exhibited higher IC50 values than the Col1 control, highlighting their greater potential as preclinical models for drug screening.

**Figure 5:**
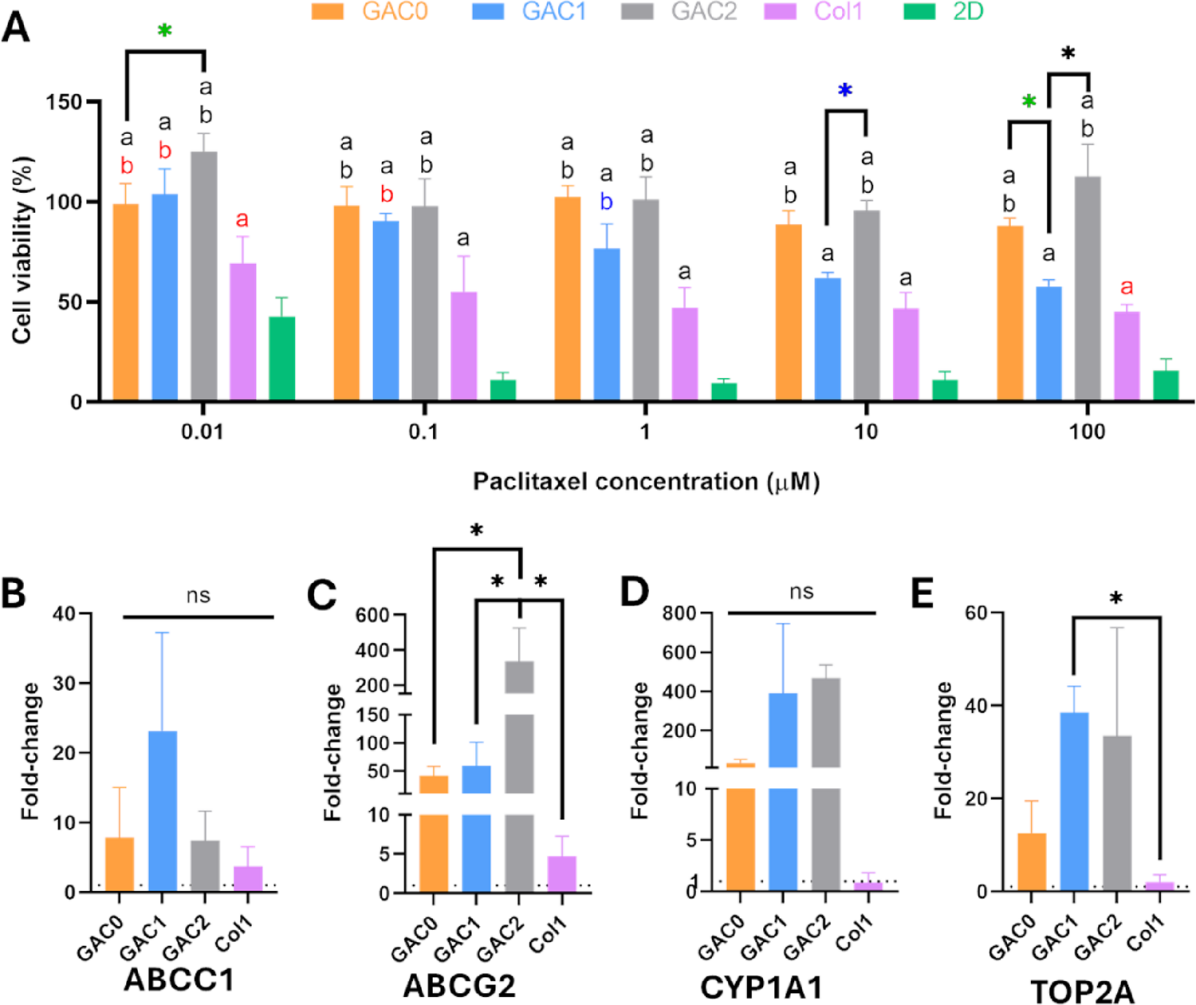
Chemotherapy resistance of BxPC-3 growing in GAC0, GAC1, and GAC2 hydrogels. (A) BxPC-3 viability in bioprinted hydrogels and Col1 and 2D controls after incubation with paclitaxel for 48 h (two-way ANOVA; *: statistical differences between bioinks, a: statistical differences with Col1 controls, b: statistical differences with 2D controls, black symbols: *p* < 0.0001, red symbols: *p* < 0.001, blue symbols: *p* < 0.01, and green symbols: *p* < 0.05). (B–E) Drug resistance gene expression by RT-qPCR: ABCC1 (B), ABCG2 (C), CYP1A1 (D), and TOP2A (E). Gene expression is expressed as 2^–ΔΔCt^ using 2D cultures as controls. (One-way ANOVA, *n* = 3, ns: no significant, and * *p* < 0.05).

To further investigate the impact of Col1 addition, we analyzed the expression of drug-resistance-related genes in BXPC-3 cells within our models using RT-qPCR. ABC multidrug transporters play a crucial role in mediating drug resistance across various cancers, including PDAC. The overexpression of these transporters is associated with tumor progression and the acquisition of a more aggressive phenotype.^89,90^ ABCC1 encodes multidrug resistance-associated protein 1 (MRP1), while ABCG2 encodes breast cancer resistance protein (BCRP). These transporters facilitate the efflux of various chemotherapeutic agents across physiological barriers, contributing to drug resistance in PCCs.^90,91^ In this study, although ABCC1 expression appeared higher in GAC1, no statistically significant differences were found between the models and the control (Figure 5B). In contrast, ABCG2 expression was notably elevated in GAC2, correlating with the increased IC50 observed in the paclitaxel resistance experiments (Figure 5C).

Tumors can also express various isoforms of cytochrome P450 enzymes, enabling the metabolism of anticancer drugs into inactive metabolites. The expression of these enzymes represents a mechanism of drug resistance, as they can counteract the effects of chemotherapeutic agents.^92^ CYP1A1 is a polymorphic variant of these enzymes that, when overexpressed in tumors, utilizes anticancer drugs as substrates, leading to their inactivation.^93^ In this study, we observed an increased expression of this enzyme in all designed models; however, no statistically significant differences were found between them (Figure 5D). Finally, we analyzed the expression of type IIA topoisomerase (TOP2A), an enzyme that induces double-strand breaks in DNA to resolve topological constraints and maintain genome stability. In cancer cells, TOP2A is often overexpressed, increasing the level of formation of TOP2A-DNA cleavage complexes. This process leads to DNA damage, accumulating mutations in tumor cells and promoting carcinogenesis.^94^ In the designed models, TOP2A expression was elevated in GAC1 and GAC2, the models containing Col1 (Figure 5E). However, only GAC1 exhibited a statistically significant increase in expression compared to that of the Col1 control.

These results indicate that the addition of Col1 to the bioinks allows for the replication of the influence of desmoplasia and ECM on PDAC behavior and development. The designed GAC1 and GAC2 models can imitate the drug resistance, desmoplasia marker expression, matrix remodeling, and mechanical properties observed on in vivo PDAC tumors. In conclusion, GAC1 and GAC2 present high potential as PDAC preclinical models for chemotherapeutic agents and drug screening.

## Conclusions

In this study, two bioinks, GAC1 and GAC2, capable of modeling the desmoplasia and TME characteristics found in in vivo PDAC tumors, were developed. PCCs proliferated within the developed tumoroids, showing spheroid formation and ECM deposition. The presence of Col1 in these bioinks effectively imitates the influence of this protein on in vivo PCCs, upregulating the expression of markers related to desmoplasia, inflammation, and drug resistance. On the whole, the developed bioinks have high potential for 3D PDAC modeling, by closely imitating human pathophysiology while addressing the limitations of current 2D and animal models. Furthermore, additive manufacturing is a versatile approach to refine the screening of chemotherapeutic agents and anticancer drugs, improve the selection of preclinical drugs for human testing, and enable high-throughput screening. Future research into the capabilities of these bioinks will further underscore the potential of these PDAC models in mimicking desmoplasia and advancing current preclinical study methodologies.

## Supplementary Material



## Notes

RT-qPCR primer sequences, ^1^H NMR spectra of gelatin and GelMA, architecture of the bioprinted scaffolds (filaments and tumoroids), and design and bioprinted scaffolds used to assess the diffusion rate and printability (PDF)
