# Supplementary material for: A 3D Bioprinted Pancreatic Cancer Model Using Collagen-Gelatin Methacrylamide-Alginate Bioinks to Mimic the Desmoplastic Microenvironment
Source: Biomacromolecules. 2025 Oct 14;26(11):7275–88. doi: 10.1021/acs.biomac.5c00450 (PMC12606650; doi:10.1021/acs.biomac.5c00450)
Supplement: Supplementary file 1 [file bm5c00450_si_001.pdf]

A 3D Bioprinted Pancreatic Cancer Model Using Collagen-Gelatin Methacrylamide-Alginate Bioinks to Mimic the Desmoplastic Microenvironment

Uxia Gato-Diaz [1,2], Sandra Blanco-Garcia [1], Diana Peixoto [1], Angel Concheiro [1,2], Carmen Alvarez-Lorenzo [1,2,], Barbara Blanco-Fernandez [1,2,*].*

[1]I+D Farma Group (GI-1645), Department of Pharmacology, Pharmacy and Pharmaceutical Technology, Facultad de Farmacia and Instituto de Materiales (iMATUS), Universidade de Santiago de Compostela, 15782 Santiago de Compostela, Spain.

[2]Health Research Institute of Santiago de Compostela (IDIS), Complexo Hospitalario Universitario de Santiago de Compostela, Travesa da Choupana s/n., 15706 Santiago de Compostela, Spain.

Table S1. RT-qPCR primers (5' to 3') sequence.

Name	Forward	Reverse
ABCC1	CCATCCACGACCCTAATCCC	ACTTGTTCCGACGTGTCCTC
ABCG2	GACTTATGTTCCACGGGCCT	GGCTCTATGATCTCTGTGGCTTT
ACTB	CACCATTGGCAATGAGCGGTTC	AGGTCTTTGCGGATGTCCACGT
BAX	TCAGGATGCGTCCACCAAGAAG	TGTGTCCACGGCGGCAATCATC
CDH1	ATTTTTCCCTCGACACCCGAT	TCCCAGGCGTAGACCAAGA
COL1A1	GATTCCCTGGACCTAAAGGTGC	AGCCTCTCCATCTTTGCCAGCA
CYP1A1	GAGGTGGTTGGCTCTGGAAA	TGTGTCTCTTGTTGTGCTGTGG
EpCAM	GCCAGTGTACTTCAGTTGGTGC	CCCTTCAGGTTTTGCTCTTCTCC
HAS2	GTCATGTACACAGCCTTCAGAGC	ACAGATGAGGCTGGGTCAAGCA
IL1B	CTCTTCGAGGCACAAGGCAC	GGCTGCTTCAGACACTTGAGC
IL6R	GACTGTGCACTTGCTGGTGGAT	ACTTCCTCACCAAGAGCACAGC
MKi67	CTTTGGGTGCGACTTGACGA	ACAACTCTTCCACTGGGACG
TGFB1	CAAGTGGACATCAACGGGTTC	GCAGCAGTTCTTCTCCGTGG
TNFR SF1A	CCGCTTCAGAAAACCACCTCAG	ATGCCGGTACTGGTTCTTCCTG
TOP2A	TGTGGAATTAGTGACCCAGCAAA	TTTGTTTGTTGTCCGCAGCA
VEGFA	CAAAAACGAAAGCGCAAGAAA	GCGGGCACCAACGTACAC
VIM	CCTCCGGGAGAAATTGCAGG	GCGTTCAAGGTCAAGACGTG
WWTR1	CTCCTTTTCGCCCAGCACTA	CCTGGCAGTCTAAGGGCTTC
YAP1	TGTCCCAGATGAACGTCACAGC	TGGTGGCTGTTTCACTGGAGCA

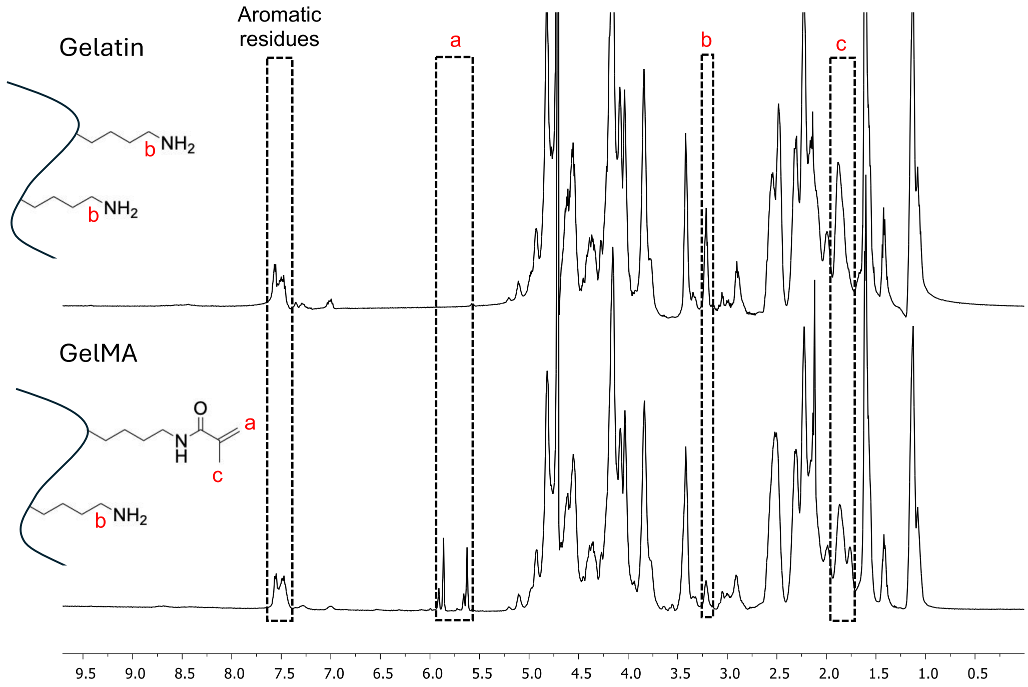

Figure S1. ^{1}H-NMR spectra of Gelatin and GelMA in D_2O. Characteristic protons of GelMA and GelMA are highlighted, being a the acrylic proton of methacrylate groups, b the methylene proton of unmodified lysine, and c the methyl protons of methacryloyl groups. The protons of the Phenylalanine aromatic ring (aromatic residues) of each ^{1}H-NMR were used for normalization.

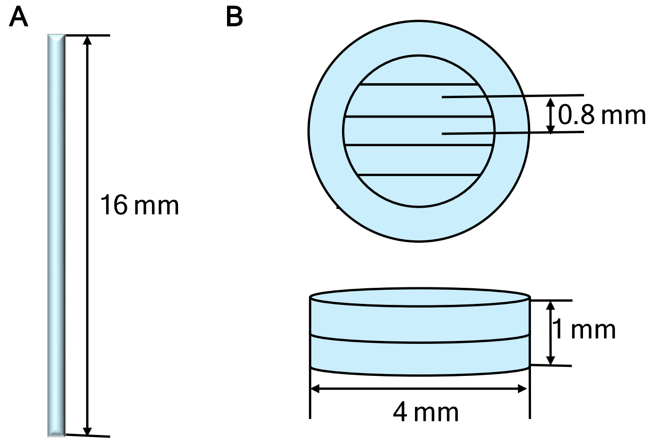

Figure S2. Architecture of the bioprinted scaffolds. (a) Filament dimensions used for assessing the shape fidelity and spreading ratio. (b) Structure of the bioprinted tumoroid hydrogels.

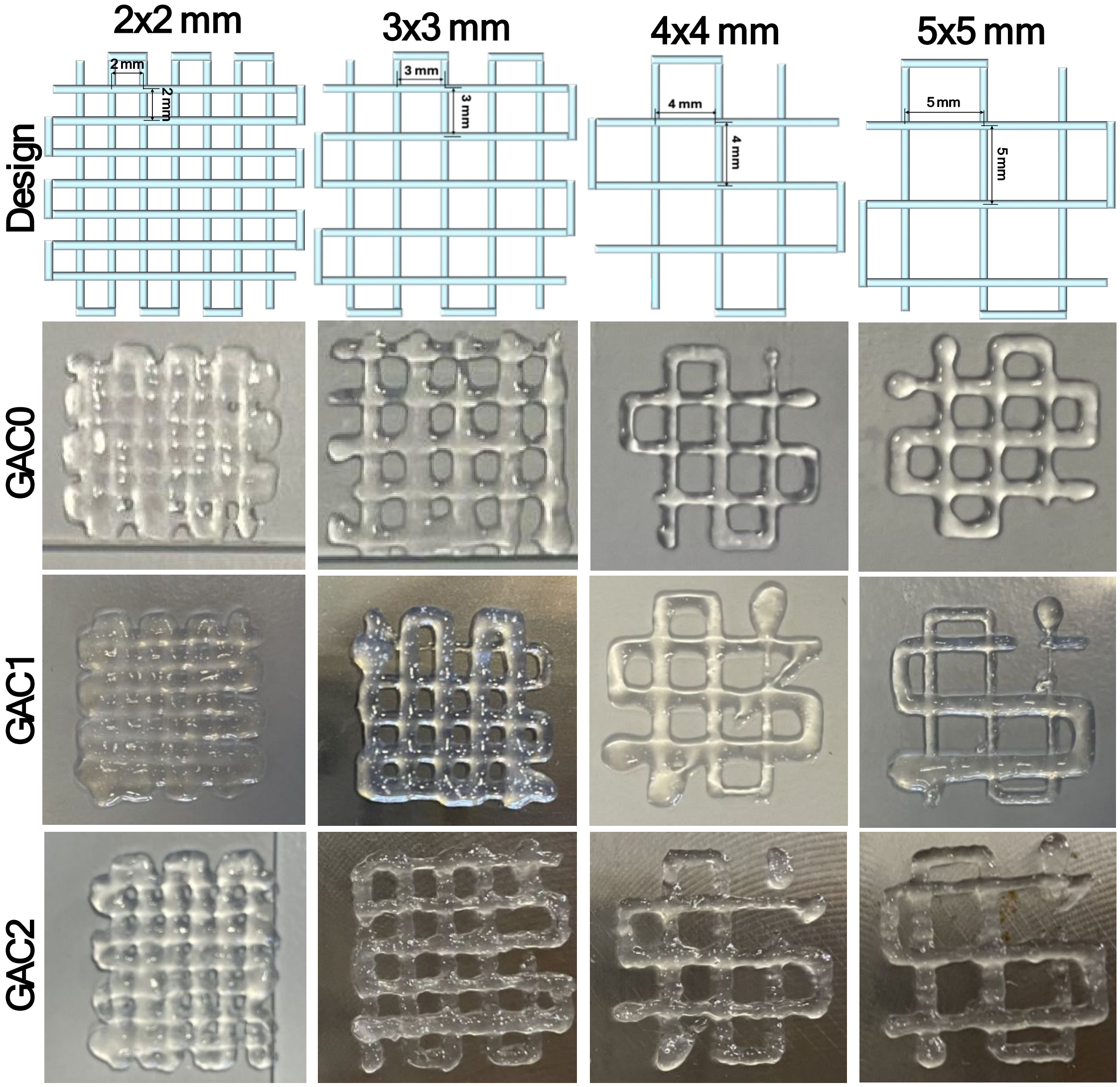

Figure S3. Design and bioprinted scaffolds with pore sizes of 3.06 mm^2 (2x2 mm), 7.56 mm^2 (3x3 mm), 14.06 mm^2 (4x4 mm), and 22.56 mm^2 (5x5 mm) were used to assess the diffusion rate and printability of the GAC0, GAC1, and GAC2 bioinks.
